# An Improved 4H-SiC MESFET with a Partially Low Doped Channel

**DOI:** 10.3390/mi10090555

**Published:** 2019-08-23

**Authors:** Hujun Jia, Yibo Tong, Tao Li, Shunwei Zhu, Yuan Liang, Xingyu Wang, Tonghui Zeng, Yintang Yang

**Affiliations:** School of Microelectronics, Xidian University, Xi’an 710071, China

**Keywords:** 4H-SiC, MESFET, simulation, PAE

## Abstract

An improved 4H-SiC metal semiconductor field effect transistor (MESFET) based on the double-recessed MESFET (DR-MESFET) for high power added efficiency (PAE) is designed and simulated in this paper and its mechanism is explored by co-simulation of ADS and ISE-TCAD software. This structure has a partially low doped channel (PLDC) under the gate, which increases the PAE of the device by decreasing the absolute value of the threshold voltage (*V*_t_), gate-source capacitance (*C*_gs_) and saturation current (*I*_d_). The simulated results show that with the increase of *H*, the PAE of the device increases and then decreases when the value of *N*_PLDC_ is low enough. The doping concentration and thickness of the PLDC are respectively optimized to be *N*_PLDC_ = 1 × 10^15^ cm^−3^ and *H* = 0.15 μm to obtain the best PAE. The maximum PAE obtained from the PLDC-MESFET is 43.67%, while the PAE of the DR-MESFET is 23.43%; the optimized PAE is increased by 86.38%.

## 1. Introduction

With the development of the semiconductor industry, SiC, diamond and GaN, the third-generation semiconductor materials, have become a research hotspot because of their high critical field strength, wide band gap and high carrier saturation rate [1,2,3,4,5,6]. 4H-SiC is used to manufacture power devices such as MESFETs due to its larger band gap and higher electron mobility compared to those of 3C-SiC and 6H-SiC [7]. Nowadays, the mainstream research direction on 4H-SiC MESFETs is to achieve better output power density by making changes to the device structure [8,9]. However, in order to achieve green development, enabling devices to have higher energy conversion efficiency has become a new central issue of research. In the papers An Improved DRBL AlGaN/GaN HEMT with High Power Added Efficiency [10] and An Improved UU-MESFET with High Power Added Efficiency [11], a higher power added efficiency (PAE) was obtained by balancing the parameters of the devices. The PAE of the improved with an ultrahigh upper gate MESFET (IUU-MESFET) and the double recessed barrier layer (DRBL) AlGaN/GaN HEMT increased 18% and 48%, respectively. In the aforementioned research works, PAE simply replaces the RF output power with the difference between output and input power in the drain efficiency equation. A larger PAE means that a larger output power can be obtained under the same input power. This is crucial for sustainable development.

In this paper, an improved 4H-SiC MESFET with a partially low doped channel (PLDC) is designed and simulated to improve the PAE of the 4H-SiC DR-MESFET [12] using ISE-TCAD and ADS. A partially low doped channel is used to balance the parameters of the device by adjusting the doping concentration and thickness. The key to this structure is to improve the AC/RF characteristics of the device and improve the PAE of the device. This ensures that the device has lower energy consumption at the same output power, which has great significance for RF power amplifier applications. In the second part of this paper, the basic features and simulation process of the PLDC-MESFET are introduced, as are the models used in the simulation. In the third section, the main impact of the PLDC on the parameters and PAE of the device is introduced and the mechanism is discussed. In the fourth section, we conclude that the PLDC is helpful for the improvement of the PAE of the DR-MESFET.

## 2. Device Structure 

**Figure 1:**
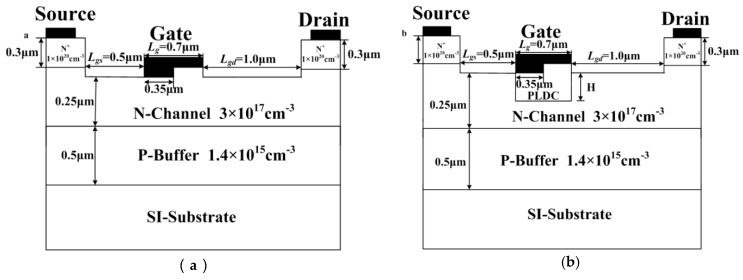
Schematic cross-sections of the (**a**) DR 4H-SiC MESFET, (**b**) partially low doped channel (PLDC) 4H-SiC MESFET.

The 2D schematic cross-sections of the DR-MESFET and PLDC-MESFET structures are shown in Figure 1a,b, respectively. The difference between the two devices is that the PLDC-MESFET has a partially low doped channel under the gate. The PLDC was realized by high-energy ion implantation and high-temperature annealing processes. It should be noted that the P-type impurity is implanted to compensate for the formation of lightly doped regions [13]. The thickness and the concentration of the PLDC are denoted as *H* and *N*_PLDC_, respectively. The *N*_PLDC_ was set to 1 × 10^17^ cm^−3^, 1 × 10^16^ cm^−3^ and 1 × 10^15^ cm^−3^. The *H* was set from 0 to 0.25 μm in a step of 0.05 μm. 

The main physics models were applied in ISE-TCAD tools simulation [14], including Mobility (Doping Dep, HighFieldSat Enormal), Effective Intrinsic Density (Band Gap Narrowing (OldSlotboom), Incomplete Ionization, Recombination (SRH (Doping Dep) and Auger Avalanche (Eparallel). The criterion of breakdown was Break Criteria {Current (Contact = “gate” Absval = 1e3)}. The main solving model was Coupled {Poisson Electron Hole}. Mobility models were used to solve the phenomenon of the mobility of carriers being degraded by many factors. Recombination models were used to calculating the lifetime of carriers. The Effective Intrinsic Density model was used to calculate the effective band gap. Incomplete Ionization must be considered, as this occurs in the case of aluminum acceptors in silicon carbide. The temperature of the simulations was 300 K. The major parameters of the device measured were saturation current (*I*_d_), threshold voltage (*V*_t_), gate–source capacitance (*C*_gs_) and transconductance (*g*_m_). Those parameters are used in ADS to modify the EE_FET3 model. The modified EE_FET3 model and “Load-Pull PAE, Output Power Contours” model [15] were used to measure the PAE of the device under the same bias conditions. The working bias conditions were set as follows: *V*_gs_ was −8.0 V, *V*_ds_ was 28 V, RF was 850 MHz and Pavs_dBm was 28 dBm. Keeping the bias condition and changing the parameters obtained from ISE-TCAD, the PAE of the device under different thicknesses and doping concentrations can be calculated as follows [16].

(1) $$\eta \left(\mathrm{PAE}\right)=\frac{P_{\mathrm{out}}-P_{\mathrm{in}}}{P_{\mathrm{dc}}}$$

where *P*_out_ is output power, *P*_in_ is input power and *P*_dc_ is DC power.

## 3. Results and Discussion

### 3.1. The Effect of Doping Concentration and Thickness On the Device Parameters 

**Figure 2:**
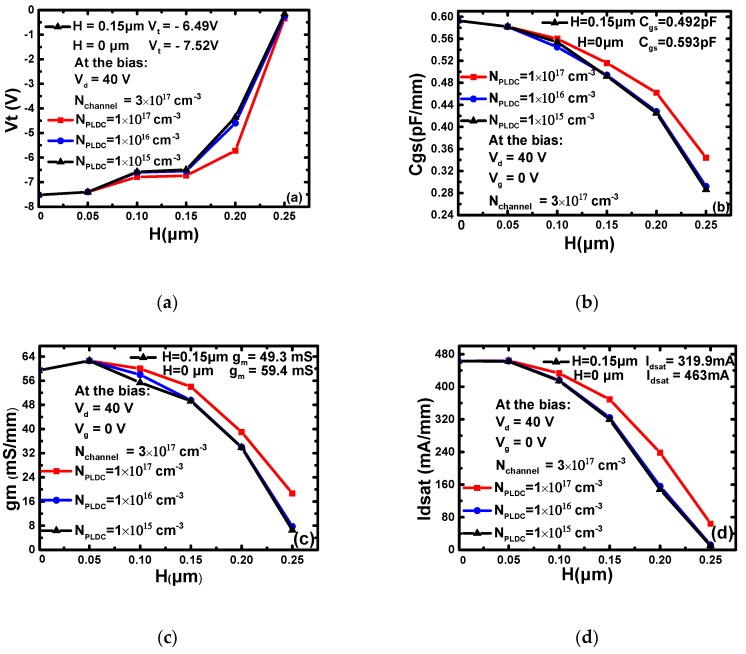
The effect of *N*_PLDC_ and *H* on the device parameters: (**a**) *V*_t_-*N*_PLDC_ and *H*, (**b**) *C*_gs_-*N*_PLDC_ and *H*, (**c**) *g*_m_-*N*_PLDC_ and *H*, (**d**) *I*_dsat_-*N*_PLDC_ and *H*.

As showing in Figure 2, the parameters of the device are greatly affected by the doping concentration (*N*_PLDC_) and thickness (*H*) of the PLDC. The effect of *N*_PLDC_ and *H* on *V*_t_ is shown in Figure 2a. With the decrease of *N*_PLDC_, the absolute value of *V*_t_ decreases obviously. When *H* increases, the *V*_t_ overall trend is also decreasing. This is because the changes in *N*_PLDC_ and *H* directly control the total carrier concentration in the channel, and *V*_t_ is proportional to the total carrier. Figure 2b shows the effects of *N*_PLDC_ and *H* on *C*_gs_. With the decrease of *N*_PLDC_ and the increase of *H*, *C*_gs_ decreases. On the one hand, the PLDC suppresses the under-gate depletion layer extending to the source side, and on the other hand, it reduces the total number of carriers in the channel, thereby reducing the input capacitance of the device. In the Figure 2c, *g*_m_ increases first and then decreases. The reason for this formation may be that the thinner low doped layer can increase the gate’s ability to control the current by inhibiting the diffusion of the depletion layer to some extent. When *H* is thick enough, the ability of the gate to control the current will be reduced. So, *g*_m_ decreases. In Figure 2d, *I*_dsat_ is roughly decreased as *H* increases and *N*_PLDC_ decreases. This is mainly caused by the decrease of the channel carrier concentration. When *H* is 0.25 μm, the parameters exhibit a sharp decrease and the DC characteristic of the device becomes poor. It is indicated by the simulation results that the PLDC-MESFET has smaller values of *C*_gs_, *g*_m_, *V*_t_ and *I*_dsat_ as compared to those of the original device.

### 3.2. The Influences of Doping Concentration and Thickness on the PAE

**Figure 3:**
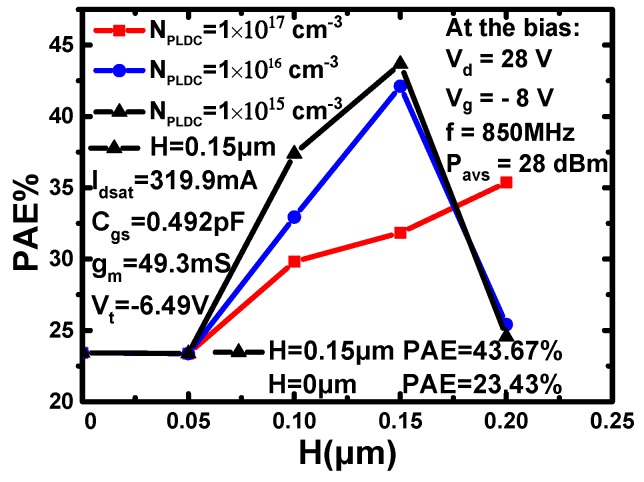
The effects of *N_PLDC_* and *H* on the PAE.

The influences of the doping concentration and thickness on the PAE are shown in Figure 3. It can be seen that when *H* is smaller than 0.20 μm, the PAE of the device increases with the decrease of *N*_PLDC_. When *H* is 0.20 μm and *N*_PLDC_ is 1 × 10^15^ cm^−3^ or 1 × 10^16^ cm^−3^, the PAE of the device decreases sharply. When *H* is 0.20 μm and *N*_PLDC_ is 1 × 10^17^ cm^−3^, the PAE of the device increases. When *H* is 0.25 μm, the simulation results show that the DC characteristics and AC characteristics of the device are poor, and the PAE of these structures is low. The maximum value of the PAE is obtained when the *N*_PLDC_ is 1 × 10^15^ cm^−3^, the *H* is 0.15 μm. The PAE of the new device is 43.67% while the PAE of the original device is 23.43%. The optimized PAE is increased by 86.38%. The PAE of the IUU-MESFET and DRBL AlGaN/GaN HEMT increase 18% and 48%, respectively. So, the PLDC has a great effect on improving the PAE of the device. In the paper *107 W CW SiC MESFET with 48.1% PAE,* the experimental PAE of the device at 2 W (33 dBm) is close to 25% [17]. The PAE of the DR-MESFET is 23.43% at 0.63 W (28 dBm). This is essentially consistent with the simulation results.

### 3.3. Mechanism Discussion

**Figure 4:**
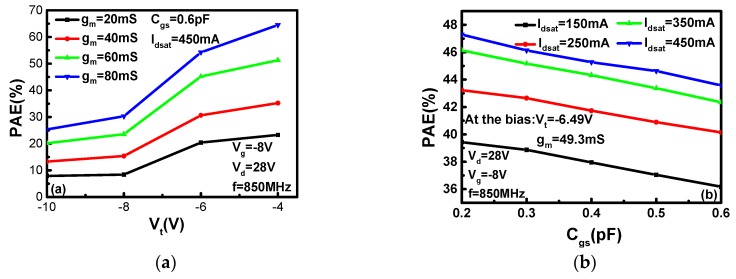
The effect of device parameters on PAE: (**a**) PAE-*V_t_* and *g_m_*, (**b**) *PAE*-*C_gs_* and *I_dsat_*.

Figure 4a,b shows the influence of the parameters on PAE at the same bias when *V*_gs_ is −8.0 V, *V*_ds_ is 28 V, RF is 850 MHz and Pavs_dBm is 28 dBm. As shown in Figure 4a, the PAE increases with the increase of *V*_t_ when *g*_m_ is a constant. When *V*_t_ is a constant, the PAE also increases with the increase of *g*_m_. When *g*_m_ is between 40 and 60 mS, the PAE of the device has the biggest change. This can be observed by the distance between the two curves. Figure 4b shows the influence of *I*_dsat_ and *C*_gs_ on the PAE. With the increase of *C*_gs_, the PAE decreases. With the increase of *I*_dsat_, the PAE increase. Furthermore, the larger *I*_dsat_ is, the slower PAE increases.

From the analysis above, it can be concluded that the smaller the absolute value of *V*_t_, the bigger the PAE, and the smaller the *C*_gs_, the bigger the PAE. For *g*_m_, a bigger *g*_m_ means a higher current gain, so it a larger output can be obtained under the same input. According to Figure 4a, the PAE is proportional to *g*_m_. This is the reason why the PAE of the device decreases sharply when *H* is 0.20 μm and *N*_PLDC_ is 1 × 10^15^ cm^−3^ or 1 × 10^16^ cm^−3^. When *H* is 0.20 μm and *N*_PLDC_ is 1 × 10^17^ cm^−3^, the PAE of the device increases because *g*_m_ is not the key factor compared with *V*_t_, *I*_dsat_ and *C*_gs_. The PAE of the device is decided by the influences of those parameters.

It can be seen that the doping concentration and thickness of the PLDC are optimized to be *N*_PLDC_ = 1 × 10^15^ cm^−^^3^ and *H* = 0.15 μm. Table 1 shows some main parameters of the two devices. It can be seen that the PAE of the PLDC-MESFET is 43.67%, which is higher than the PAE of 23.43% of the DR-MESFET. Compared the two devices, the PLDC-MESFET has a smaller threshold voltage, smaller input capacitance, smaller transconductance and smaller saturation current than the DR-MESFET. The increase of the PAE is influenced by the combination of these parameters. When the absolute value of *V*_t_ decreases, the device is easier to turn on and gains a larger output current. So, the output power *P*_out_ increases and a higher PAE is reached. According to Formula (2) [16], a smaller input capacitance *C*_gs_ means the device has less energy loss when working in RF (charging and discharging).

(2) $$P_{\mathrm{dyn}}=E_{\mathrm{VD}}-E_{c}=\int_{0}^{\infty }i_{\mathrm{vd}}(t)V_{d}dt-\int_{0}^{\infty }i_{\mathrm{vd}}(t)v_{\mathrm{out}}dt=CV_{D}{}^{2}-\frac{CV_{D}^{2}}{2}=\frac{CV_{D}^{2}}{2}$$

where *P*_dyn_ is the dynamic power consumption flipped once, *E*_VD_ is the energy obtained from the power source, *E*_c_ is the capacitor stored energy, *C* is the gate–source capacitor and *V*_D_ is the drain voltage. A small *C*_gs_ also increases the input impedance of the device. Therefore, *P*_out_ of the device increases and *P*_in_ decreases. For *I*_dsat_, a small *I*_dsat_ indicates a small *P*_out_. Under the influence of these parameters, the device has a big PAE. In there, *g*_m_ is sacrificed to obtain a higher PAE. Though a larger *g*_m_ is helpful to increase PAE, the influences of the other parameters on PAE are more obvious. So, the maximum value of PAE is 43.67% when *N*_PLDC_ is 1 × 10^15^ cm^−3^ and *H* is 0.15 μm, as obtained by sacrificing some of the DC performances of the device.

## 4. Conclusions

An improved 4H-SiC MESFET with a partially low doped channel is designed and simulated in this paper to increase the PAE of the device. The results show that the maximum PAE of the PLDC-MESFET is 43.67%, while the PAE of the DR-MESFET is 23.43%; the optimized PAE was increased by 86.38%. A way to design an energy efficient amplifier is proposed in this paper by balancing the parameters of the device. This ensures that the device has lower energy consumption at the same output power, which has great significance for RF power amplifier applications.

## Figures and Tables

**Table 1:** Comparison of performance parameters of the two structures.

Parameters	DR 4H-SiC MESFET	PLDC 4H-SiC MESFET
*I*_dsat_ (mA/mm)	448.00	319.90
*V*_b_ (V)	125.35	130.20
*g*_m_ (mS/mm)	59.30	49.30
*V*_t_ (V)	−7.52	−6.49
*C*_gs_ (pF/mm)	0.59	0.49
PAE (%)	23.43	43.67
